# Copper (IV) Stabilization in Macrocyclic Complexes with 3,7,11,15-Tetraazaporphine, Its Di[benzo]- or Tetra[benzo] Derivatives and Oxide Anion: Quantum-Chemical Research

**DOI:** 10.3390/ma13143162

**Published:** 2020-07-15

**Authors:** Oleg V. Mikhailov, Denis V. Chachkov

**Affiliations:** 1 Department of Analytical Chemistry, Certification and Quality Management, Kazan National Research Technological University, K. Marx Street 68, Kazan 420015, Russia; 2 Kazan Department of Joint Supercomputer Center of Russian Academy of Sciences–Branch of Federal Scientific Center “Scientific Research Institute for System Analysis of the RAS”, Lobachevskii Street 2/31, Kazan 420111, Russia; de2005c@gmail.com

**Keywords:** copper(**IV**), oxide anion, 3,7,11,15-tetraazaporphine, benzo-derivatives, DFT method

## Abstract

Using the data of a quantum chemical modeling of molecular structures obtained by the density functional theory (DFT), the possibility of the existence of a copper macrocyclic complexes with 3,7,11,15-tetraazaaporphine, *trans*-di[benzo] 3,7,11,15-tetraazaaporphine or tetra[benzo] 3,7,11,15-tetraazaaporphine and oxide anion where oxidation state of copper is **IV**, was shown. The values of the parameters of molecular structures and NBO analysis for such complexes were presented, too.

## 1. Introduction

**Figure 1:**
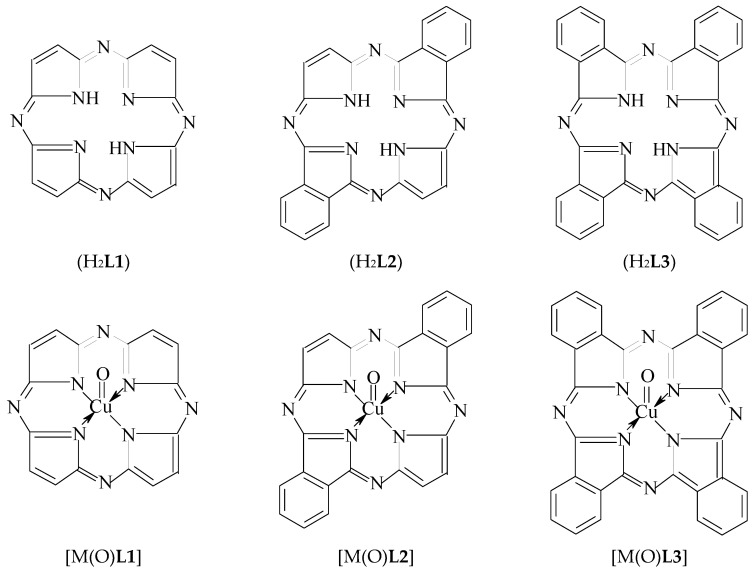
Structural formulas of macrocyclic ligands H_2_**L1,** H_2_**L2** and H_2_**L3** and copper complexes formed by them and oxo-anions 2. Calculation Method.

Before [1], using a modeling by the density functional theory (DFT) method, we shown the possibility in principle to stabilize the copper(**IV**) oxidation state that is unusual for the given element, in a heteroligand chelate complex containing a (NNNN)-donoratomic tetradentate macrocyclic ligand–3,7,11,15-tetraazaporphine (porphyrazine) (H_2_**L1**) located in the "equatorial" plane with N4 macrocycle and two fluoride ligands (F^−^) occupying axial positions (i.e., the vertices) of this coordination polyhedron. As it is known, such macrocyclic ligands as porphine and its numerous derivatives, can stabilize the various oxidation degrees (states) of *d*-elements, low as well as high (see, for example, [2,3,4,5,6]). In this connection, it can be expected that to stabilize such high oxidation state of copper as **IV**, in principle, 3,7,11,15-tetraazaporphine as well as its various derivatives, in particular, *trans*-di[benzo]3,7,11,15-tetraazaporphine (H_2_**L2**) and tetra[benzo]3,7,11,15-tetraazaporphine (tetra[benzo]porphyrazine, phthalocyanine) (H_2_**L3**), may be able. On the other hand, fluoride ion is not only ligand capable of stabilizing Cu(**IV**) in complexes together with the macrocyclic ligands indicated above; there is also another ligand which can stabilize high oxidation states of various *d*-elements, namely the oxide anion O^2−^ [7,8]. By taking into account the aforesaid, it seems appropriate to use for the Cu(**IV**) oxidation state stabilization, namely the combination of any of the macrocyclic ligands indicated above, with oxide anion, that takes place in [M(O)**L1**], [M(O)**L2**] and [M(O)**L3**] complexes (where **L1**^2−^, **L2**^2−^ and **L3**^2−^ is double deprotonated form of 3,7,11,15-tetraazaporphine, *trans*-di[benzo] 3,7,11,15-tetraazaporphine and tetra[benzo]3,7,11,15-tetraazaporphine, respectively) (see Figure 1). Currently, any information about such a synthesis and physicochemical characteristics of the given macrocyclic complexes is absent in the literature, but now, the possibility of existence of these compounds can be estimated by using modern quantum-chemical calculation methods. However, until now, quantum chemical calculations of these complexes have not been performed a period, too. In connection with the foregoing, the given article is devoted to consideration of possibility of the existence of [Cu(O)**L1**], [Cu(O)**L2**] and [Cu(O)**L3**] complexes using one of version of DFT quantum-chemical calculation method which is most popular and, at the same time, rather reliable method in the modern quantum chemistry.

Quantum-chemical research of complexes indicated above, were done by using DFT method with OPBE/TZVP level, where the common TZVP extended triple zeta split-valence basis set [9,10] and the OPBE nonhybrid functional [11,12] were combined. The given method for the 3*d* element compounds allows to obtain most adequately relative energy stabilities of high-spin and low-spin states, and, also, reliably to find most important geometric parameters of their molecular structures [12,13,14,15,16]. This method was used by us in a number of our earlier works, for example, in [17,18,19,20,21]. By using the given method, we found that the calculated geometric parameters of the molecular structures of macrocyclic compounds formed by various 3*d*-elements with tetra[benzo] porphyrazine (H_2_**L3**) (and, in particular, for copper complex), only very slightly differ from the corresponding experimental values. The calculation ns were carried out using the Gaussian09 program package [22]. The results of quantum chemical calculations were visualized by using the *ChemCraft* software (Version 1.8, Wallingford, CT, USA). In all cases, the correspondence of the found stationary points to energy minima was proved by the calculation of second derivatives of energy with respect to atom coordinates; besides, any of equilibrium structures which corresponds to minima of the potential energy surfaces, had only positive value of frequency. Theoretically, copper in the oxidation state **IV** must have 3*d*^7^ electronic configuration; that is why, only spin multiplicities 2 and 4 were considered in calculation. In addition, during our calculations, both the atomic motion and the electron motion were taken into account, which made it possible to track the possibility of internal electron transfer with the formation of an “oxidized” macrocycle. Among the structures optimized at the multiplicities indicated, the lowest-lying structure was selected. Parameters of molecular structures with these multiplicities were calculated by the unrestricted (*UOPBE*) method. A calculation of standard thermodynamic parameters of formation of metal complexes under study was carried by using methodology described in [23].

## 2. Results

**Figure 2:**
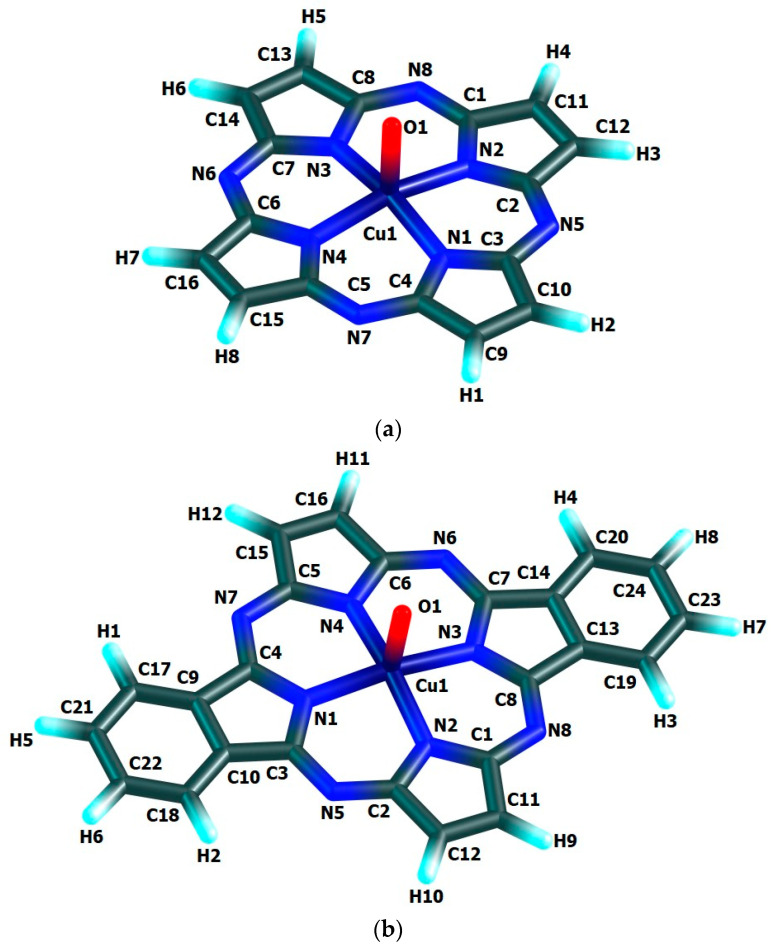

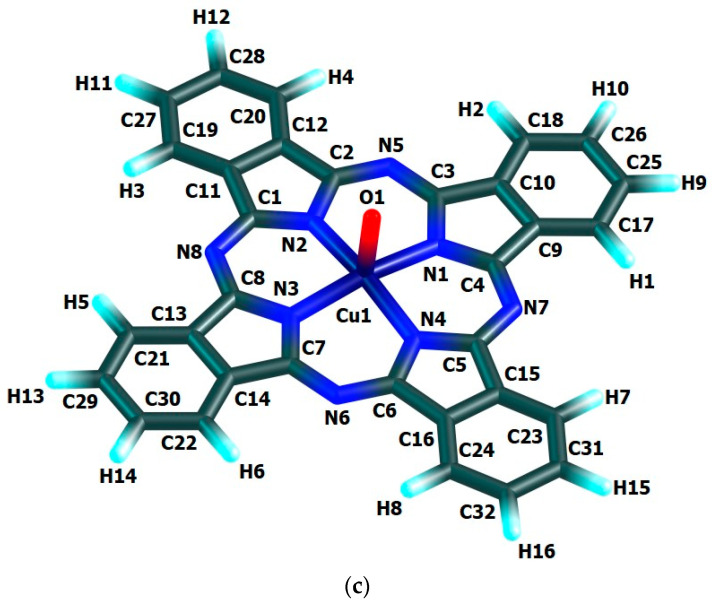
Molecular structures of Cu(IV) macrocyclic compounds of types (**a**) [Cu(O)**L1**] (ground state–spin doublet, *M_S_* = 2), (**b**) [Cu(O)**L2**] and (**c**) [Cu(O)**L3**] (ground state–spin quartet, *M_S_* = 4) found using DFT OPBE/TZVP method.

**Figure 3:**
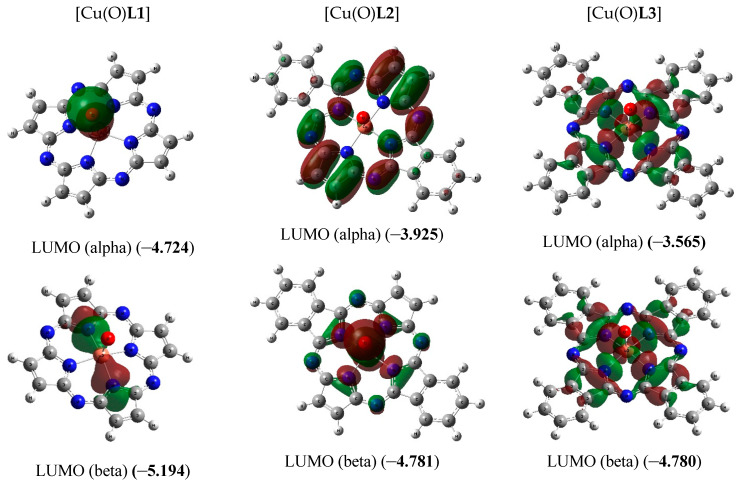

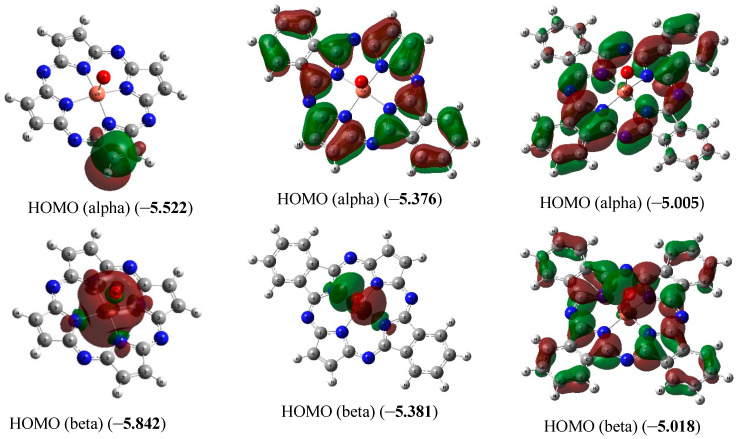
Images of highest occupied (HOMO) and lowest unoccupied (LUMO) molecular orbitals in the [Cu(O)**L1**] (ground state–spin doublet, *M_S_* = 2) [Cu(O)**L2**] and [Cu(O**)L3**] complexes (ground state–spin quartet, *M_S_* = 4). The values of energies of these molecular orbitals (in brackets) are given in eV. The symbol “alpha” corresponds to electron with spin (+1/2), “beta”, to electron with spin (–l/2).

The chemical bond lengths between atoms and bond angles in the macrocyclic complexes under study calculated by DFT method level indicated above, are presented in Table 1. The molecular structures obtained as a result of quantum-chemical calculation by this method are shown in Figure 2, the images of highest occupied (HOMO) and lowest unoccupied (LUMO) molecular orbitals, in Figure 3.

Electric moments of dipole for these chemical compounds calculated by DFT method are 2.26 ([Cu(O)**L1**]), 1.76 ([Cu(O)**L2**]) and 1.75 ([Cu(O)**L3**]) Debye units.

The ground state of [Cu(O)**L1**] in the framework of the DFT method used by us, is a spin doublet; of [Cu(O)**L2**] and [Cu(O)**L3**] complexes, is a spin quartet.

According to NBO analysis data, in the macrocyclic compounds considered here, very high degree of delocalization of electrons takes place (the charge on Cu atom is +0.8173 ē in the [Cu(O)**L1**], +0.7705 ē in the [Cu(O)**L2**] and +0.7207 ē in the [Cu(O)**L3**]). Simultaneously, the charge on O atoms is −0.5090 ē (in [Cu(O)**L1**]), −0.2047 ē ([Cu(O)**L2**]) and −0.2866 ē ([Cu(O)**L3**]). The value of <S**2> is 0.7739, 3.7625 and 3.7619, respectively; they correspond to availability of one (in [Cu(O)**L1**]) and three ([Cu(O)**L2**] and [Cu(O)**L3**]) unpaired electrons in the molecules of complexes indicated above.

The standard thermodynamic parameters of formation (Δ*H*^0^*_f_*_, 298_, *S*^0^*_f_*_, 298_ and Δ*G*^0^*_f_*_, 298_) calculated by us for the copper complexes considered here, are presented in Table 2.

## 3. Discussion

The quantum-chemical calculation data presented above, evidence that the copper(**IV**) compounds having [M(O)**L1**], [M(O)**L2**] and [M(O)**L3**] compositions and considered here, may exist, at least in the gas phase. The data in the Table 1 and, also, the images of molecular structures presented on Figure 2, allow us to make a conclusion that chelate nodes CuN_4_ are not planar whereas N_4_ groupings in them, are strictly flat since values of the sum of the angles between corresponding nitrogen donor atoms in each of such macrocyclic compounds are equal to 360°. It should be noticed in this connection that, none of four 6-membered metal chelate rings in the given compounds, are planar, too, but all four 5-membered non-chelate rings containing one N atom and four C atoms and adjacent to 6-membered ones, in the macrocyclic compounds under examination, are a strong flat configuration. This conclusion follows from the fact that BAS^5^ and BAS^6^ values in each of such closed structures is 540.0° and 720.0°, respectively (Table 1). Moreover, all 6-membered and 5-membered rings are completely identical to each other because the lengths of bonds between the corresponding atoms, and in the assortment of bond angles in them are fully identical. Nevertheless, BAS values in the CuN_4_ chelate node in the each of the given complexes is little different from 360° (not more than 5°). Hence, in the each of them, tetragonal-pyramidal orientation of the N donor atoms relative to the central copper atom with a slight deviation of it from the plane formed (NNNN) atoms, takes place. The (N–Cu–N) bond angles in the CuN_4_ grouping in the each of the complexes considered are equal between themselves (wherein, each of these angles is lesser 90°), although in [Cu(O)**L1**], [Cu(O)**L2**] and [Cu(O)**L3**] complexes these angles are slightly different (Table 1). In contrast, the (OCuN) angles formed by the oxygen, copper and any of the nitrogen atoms of the CuN_4_ atom grouping of these complexes, as a rule, are equal between themselves only in pairs. In principle, the circumstance noted is perfectly expected since Cu–N bond lengths in any of the given complexes are identical only in pairs, too. The only exception is [Cu(O)**L2**] complex where all these angles are equal between them (Table 1). Unlike the bond angles in the CuN_4_ grouping, each of (OCuN) angles is more than 90°. Hence, the displacement of copper atoms outside the plane of the donor nitrogen atoms (although it is insignificant) takes place in the each of the given complexes. There is not a center of symmetry in these macrocyclic compounds and, hence, an electric moment of the dipole for the each of them must be different from zero. However, since each of these complexes has at least three symmetry elements-one second-order axis and two symmetry planes (and [Cu(O)**L1**] and [Cu(O)**L3**]-one fourth-order axis, four second-order axes and four planes symmetry), we can assume that these values will not be too large. The values of the given parameter for the complexes under consideration (2.26, 1.76 and 1.75 Debye units for [Cu(O)**L1**], [Cu(O)**L2**] and [Cu(O)**L3**], respectively), are in good agreement with such an assumption.

As it was noted above, the ground state of copper complexes with oxo ligand and deprotonated form of **L2**^2−^ and **L3**^2−^ macrocyclic ligands in the framework of the DFT method used by us, is a spin quartet. Herewith, the nearest excited doublet state in the case of these complexes has much higher energy (50.2 and 14.6 kJ/mol, respectively). However, for the simplest of these compounds, and, namely copper complex with oxo ligand and deprotonated form of **L1**^2−^, DFT OPBE/TZVP method gives spin doublet as ground state, and nearest excited quartet state has energy 92.2 kJ/mol more compared with energy of doublet ground state. As can be seen from the data those presented in Figure 3, the difference in the energies of HOMO (alpha) and HOMO (beta) for [Cu(O)**L1**] (0.32 eV) is much larger than the similar difference for [Cu(O)**L2**] and [Cu(O)**L3**] (0.005 and 0.013 eV, respectively). Owing to that, in the case of [Cu(O)**L1**], the first Hund rule “works” for a smaller number of molecular orbitals [in other words, seven 3*d* electrons from Cu(IV) (electron configuration of which is 3*d*^7^) occupy a smaller number of MO] than in the cases of [Cu(O)**L2**] and [Cu(O)**L3**]. As a result, the low-spin ground state (spin doublet) is realized in the first of these complexes, while the high-spin (spin quartet) state is realized in the other two ones. In this regard, we note that the energies of HOMO and LUMO in [Cu(O)**L1**] as a whole are significantly lower than the energies of the analogous HOMO and LUMO in [Cu(O)**L2**] and [Cu(O)**L3**]. In addition, very significant difference between the external forms of both HOMO and LUMO in the [Cu(O)**L1**] complex, on one hand, and in the [Cu(O)**L2**] and [Cu(O)**L3**] complexes, on the other hand, takes place (Figure 3). It should be noted in this connection that testing the wave functions of the ground state for stability using the STABLE = OPT procedure showed that, in all cases (i.e., with *M_S_* = 2 and *M_S_* = 4 for the each of three complexes under examination), the wave function is stable under the perturbations considered.

A priori, for these complexes, a situation in which an internal electron transfer from a macrocycle to a copper atom with the formation of Cu(**III**) is possible, is not ruled out. Something similar is indicated, in particular, in the publications [24,25,26], according to which, in iron complexes with porphyrin ligands, where oxidation state **IV** (or even **V**) could be attributed to the Fe atom, the presence of Fe(**III**) and “oxidized” macrocycle takes place in reality. Our calculations where both the atomic motion and the electron motion were taken into account, showed that such a transfer does not occur in any of complexes under examination, and those structures that are given in our article in Figure 2, have the minimal energies. Indirect evidence for this is also the result of NBO analysis, according to which the values of <S**2> for these copper complexes are 0.7739, 3.7625 and 3.7619, respectively, which corresponds to the availability of one unpaired electron in the Cu atom in the first of these complexes and three unpaired electrons in the other two ones, which is possible if a given atom has an odd number of electrons, and therefore, the oxidation states of Cu(**IV**), but not Cu(**III**).

The all standard thermodynamic parameters of formation Δ*H*^0^*_f_*_, 298_, *S*^0^*_f_*_, 298_ and Δ*G*^0^*_f_*_, 298_, for the copper complexes considered here, according to the data of our calculation have positive values (Table 2). Therefore, any of them cannot be obtained from simple substances formed by those chemical elements that are contained in their composition (i.e., C, N_2_, O_2_ and Cu). Despite this circumstance, the data obtained by us as a result of DFT calculation, unequivocally indicate that the molecular structures and the full totality of geometric parameters of macrocyclic compounds under examination, are such that each of them can exist as a single whole. In this connection, there are certain reasons to assert that copper complexes having the compositions indicated above, are capable of independent existence, though in the gas phase.

## 4. Conclusions

Hence, the data obtained using the DFT method with OPBE/TZVP level and presented above in the given article, unambiguously predicted the possibility of the existence of three novel Cu(**IV**) complexes of types [Cu(O)**L1**], [Cu(O)**L2**] and [Cu(O)**L3**] where **L1**^2−^, **L2**^2−^ and **L3**^2−^ is double deprotonated form of 3,7,11,15-tetraazaporphine, *trans*-di[benzo]3,7,11,15-tetraazaporphine and tetra[benzo] 3,7,11,15-tetraazaporphine, respectively. In the each of them, there are four bonds formed by copper atoms-with-atoms having greater electronegativity in comparison with it, according to the exchange mechanism– two chemical bonds with nitrogen atoms and two ones with oxygen atom. According to the generally accepted definition of the term “oxidation degree”, as it was indicated in the article [21], the oxidation degree of the each of central atoms in the [Cu(O)**L1**], [Cu(O)**L2**] and [Cu(O)**L3**] complexes (i.e., Cu) is +4. In addition, since the oxidation state of any chemical element with a positive oxidation degree is determined as the modulus of the given parameter and is displayed by the corresponding Roman numeral, in each of them, the oxidation state of copper is, namely IV. Another thing is that the *real* charge on the Cu atoms in coordination compounds considered in this article, differs significantly from the value of +4.00 ē, but such a parameter has not been connected with the above definition and, if so, then in principle it cannot be used for definition of the oxidation state [7]. However, be that as it may, the results of our quantum-chemical calculation completely fit into the idea of a fairly high stability of those chemical compounds with a high oxidation state of the central atom, where the coordination polyhedron is formed from the most durable and difficult to oxidize acid ligands having atoms with the highest electronegativity (F^−^, O^2−^).

In this connection, now it is important to confirm existence of these macrocyclic metal complexes experimentally because their synthesis may be important for further development of chemistry of highest oxidation states of chemical elements at all and copper(IV) chemistry in particular. Predicting the very possibility of the existence of such complexes and modeling their molecular structures using modern quantum chemical calculations (primarily using the DFT method) is a very useful tool in solving problems associated with this synthesis. The data of such calculations also make a certain contribution to the understanding of the general problem of stabilization of the higher oxidation states of d-elements, since the obtained parameters characterizing the distribution of electron density within the entire molecular structure, directly correlate with oxidation states.

As for the possible way to obtain such complexes, in principle, each of them could be obtained by exposure to very strong oxidizing agents, for example F_2_, OF_2_ or KrF_2_, on copper(**II**) complexes with the macrocyclic ligands **L1**, **L2** and **L3**, according to the general schemes (1–3) indicated below [here **ML** (by the first letters of words Macrocyclic **L**igand) is any of **L1**, **L2** or **L3** macrocyclic ligands contained in complexes considered by us]

[Cu**ML**] + F_2_ + 2KOH→[Cu(O)**ML**] + H_2_O + 2KF (1)

[Cu**ML**] + KrF_2_ + 2KOH→[Cu(O)**ML**] + Kr + H_2_O + 2KF (2)

[Cu**ML**] + OF_2_ + 2KOH→[Cu(O)**ML**] + H_2_O + 2KF (3)

Moreover, it is not excluded that, these complexes, if they will be discovered, can be of not only purely academic, but also very substantial practical interest and may be promising in a number of industries, in particular in catalysis, photonics, electronics and electrochemical technologies. Finally, the Cu(IV) complexes under consideration may be used as “precursors” in the various reactions of synthesis coordination compounds and, also, in the reactions of coordinated ligands owing to which, in principle, novel macrocyclic compounds may be obtained.

## Figures and Tables

**Table 1:** Key bond lengths and bond angles in the [Cu(O)**L1**], [Cu(O)**L2**] and [Cu(O)**L3**] complexes.

Structural Parameter	[Cu(O)L1]	[Cu(O)L2]	[Cu(O)L3]
Cu–N bond lengths in chelate node, pm			
Cu1N1	195.5	198.0	198.2
Cu1N2	196.4	195.2	197.5
Cu1N3	195.5	198.0	198.2
Cu1N4	196.4	195.2	197.5
Bond angles in chelate node CuN_4_, *deg*			
(N1Cu1N2)	88.9	89.3	89.3
(N2Cu1N3)	88.9	89.3	89.3
(N3Cu1N4)	88.9	89.3	89.3
(N4Cu1N1)	88.9	89.3	89.3
Bond angles sum (BAS), *deg*	355.6	357.2	357.2
Nonbond angles between N atoms in N_4_ grouping, *deg*			
(N1N2N3)	89.9	90.8	90.1
(N2N3N4)	90.1	89.2	89.9
(N3N4N1)	89.9	90.8	90.1
(N4N1N2)	90.1	89.2	89.9
Nonbond angles sum (NBAS), *deg*	360.0	360.0	360.0
Bond angles in a 6-numbered chelate ring (Cu1N1C4N7C5N4), *deg*			
(Cu1N1C4)	125.8	125.1	125.1
(N1C4N7)	127.8	128.0	127.9
(C4N7C5)	122.2	122.6	123.1
(N7C5N4)	127.7	127.8	128.1
(C5N4Cu1)	125.6	126.0	125.2
(N4Cu1N1)	88.9	89.3	89.3
Bond angles sum (BAS^6^), *deg*	718.0	718.8	718.7
Bond angles in a 5-numbered ring (C3N1C4C9C10), *deg*			
(C3N1C4)	107.7	109.2	109.1
(N1C4C9)	109.2	109.0	109.1
(C4C9C10)	107.0	106.4	106.4
(C9C10C3)	106.9	106.4	108.4
(C10C3N1)	109.2	109.0	109.0
Bond angles sum (BAS^5^), *deg*	540.0	540.0	540.0
C–N bond lengths in a 6-numbered chelate ring (Cu1N1C4N7C5N4), *pm*			
N1C4	136.7	136.8	136.8
C4N7	132.8	132.3	132.3
N7C5	132.8	132.8	132.3
C5N4	136.5	136.8	137.0
C–C bond lengths in a 5-numbered ring (C3N1C4C9C10), *pm*			
C4C9	145.3	146.0	145.7
C9C10	135.9	140.6	140.6
C10C3	145.3	146.0	147.7
Cu–O bond lengths, *pm*			
Cu1O1	193.1	198.0	198.0
Bond angles between oxygen, copper and nitrogen atoms, *deg*			
O1Cu1N1	97.6	96.5	96.6
O1Cu1N2	98.5	96.5	95.9
O1Cu1N3	97.6	96.5	96.6
O1Cu1N4	98.5	96.5	95.9

**Table 2:** Standard thermodynamic parameters of formation (Δ*H*^0^*_f_*_, 298_, *S*^0^*_f_*_, 298_ and Δ*G*^0^*_f_*_, 298_) for the Cu(IV) complexes having [Cu(O)**L1**], [Cu(O)**L2**] and [Cu(O)**L3**] composition.

Complex	Δ*H*^0^*_f_*_, 298_, kJ/mole	*S*^0^*_f_*_, 298_, J/mole K	Δ*G*^0^*_f_*_, 298_, kJ/mole
[Cu(O)**L1**]	863.6	749.6	1062.9
[Cu(O)**L2**]	763.4	945.8	981.8
[Cu(O)**L3**]	718.9	1139.1	957.4
